# Alternatives to the statistical mass confusion of testing for “no effect”

**Published:** 2025-04-07

**Authors:** Josh L. Morgan

**Affiliations:** Washington University in St. Louis, Department of Ophthalmology and Visual Sciences, Neuroscience, Biology and Biomedical Science.

## Abstract

In cell biology, statistical analysis means testing the hypothesis that there was no effect. This weak form of hypothesis testing neglects effect size, is universally misinterpreted, and is disastrously prone to error when combined with high-throughput cell biology. The solution is for analysis of measurements to start and end with an interpretation of effect size.

Biologists collect measurements that can be used to inspire, refine, or distinguish between models of the world. We can use descriptive statistics to extract simplifying parameters from these measurements. We can then use statistical models to understand the precision of our measurements. Rigorous reporting of data, therefore, should include two classes of information: *magnitude* (mean, median, correlation coefficient, etc.) and *precision* (standard error, confidence interval, compatibility interval).

Statistical analysis can also provide a third class of information, quantification of the difference between the observed result and a statistical model of a hypothesis. To perform this quantification, a researcher must choose a statistical model that describes the variance of the measure of interest and an experimental sampling procedure. This statistical model can be a readily parameterized set of normal distributions or a complex computer simulation. The statistical model allows the researcher to ask, what kind of results should I expect to see if this hypothesis were true.

To quantify the relationship between a modeled hypothesis and observed results, most cell biologists ask how often their observed result, or result more different from the hypothesis being tested, is produced by the model. This fraction of the predicted results is the p-value^1^. P = 0.01 can be interpreted as follows: 1) If the assumptions of the statistical model exactly fit the experiment and 2) if the test hypothesis exactly describes the physical process, then 3) a result at least as different from the test hypothesis as the observed result would be expected in 1% of an infinite number of similar experiments. A low p-value (<0.05 by convention) is interpreted as evidence against the null hypothesis. When rigorously understood and executed, null hypothesis significance testing (NHST) can be a useful tool for making decisions with data ^2 3^. However, p-values make sense for only a narrow range of cell biology questions and experimental designs. The ubiquitous application and misinterpretation of p-values has led to a crisis in our ability to quantify data.

Problems with the interpretation of NHST^2,4–10^ have plagued it since it was popularized by R. A. Fisher a hundred years ago. These misinterpretations range from relatively subtle philosophical points about the meaning of probability to gross logical errors such as claiming large p-values as evidence that the null hypothesis is true^11,12^. Statisticians have produced a century’s worth of clarifications, admonitions, and alternative approaches in hopes of correcting these mistakes^13–17^. Many fields, including psychology^18–21^ and epidemiology ^5,22^, have responded to these issues with decades of active debate have reformed both how experiments are designed and how data is interpreted. In contrast, biologists have been relatively slow to respond to the critique of NHST. For example, from 1980 to 2020, the number of *Journal of Neuroscience* papers using p-values increased from 35% to 98%^2^.

Among the various problems with how p-values are used, there is one convention that fundamentally undermined our ability to learn from data. Cell biologists universally define the null hypothesis as the hypothesis that there was no effect^23^. In Jacob Cohen’s words, the null hypothesis became the nil hypothesis^24^. Most often, the no-effect hypothesis is the proposition that the control and experimental groups are random samples from the same population (control mean = experimental mean). There is no special reason beyond convenience to test the nil hypothesis as opposed to a hypothesis that there was some difference. However, this convenience has been a powerful force. Cell biologist have grown to believe that purpose of data quantification is to answer the non-quantitative question of effect or no-effect.

It may seem reasonable to ask whether an experimental treatment had an effect, but there is a difference between the hallway definition of “no-effect” and the statistical definition of “no-effect”. Imagine asking a colleague if the blood pressure drug they tested had an effect. Their experience will tell them that a reduction in blood pressure from 150 mmHg to 149.9 mmHg is not clinically relevant while a reduction to 125 mmHg could be life saving. If they excitedly tell you that the drug had an effect, you can assume the reduction wasn’t by 0.1 mmHg. The same isn’t true of a t-test. If you ask the t-test if the difference is zero, the p-value only tells you how often the observed difference in means (or more extreme differences) would occur if the true effect size is *exactly* zero.

## Rejecting the no-effect hypothesis tells us about the experiment, not the biology.

In a highly interconnected network like a living organism, the proposition that one component is perfectly independent of another component is trivially false. By virtu of being part of the same organism, all molecular pathways and cells can be assumed to be either directly or indirectly connected. Detecting the connection might require extremely sensitive equipment and many samples, but the biological question is never “Is there a connection?” The meaningful question is always “How strong is the connection?”.

The second problem with the no-effect hypothesis is that all experiments can be assumed to have some sampling bias ^16^. For instance, it is now recognized that circadian rhythms have detectable effects on most cellular processes. How much of the published biological literature has strong controls for time of day? The imperfections in experiments don’t have to be large to undermine the no-effect hypothesis. They only need to be imperfections.

Finally, the statistical models underpinning NHST are mathematical simplifications of biological processes. Using these models to ask how much bigger the observed effect size is than the range of effects predicted by a given model is a great way to make sense of data (Supplementary Method 1). However, there should be no expectation that any statistical model can perfectly predict the distribution of experimental results^17^.

The upshot of these three sources of guaranteed deviations from the nil hypothesis is that p-values for ALL no-effect tests will become infinitesimally small as sample sizes approach infinity. Imagine you have a dial that increases the sample size in all publications. As you turn the dial up, asterisks begin appearing over every plot and bar graph. You can keep turning the knob until each bar has a string of asterisks that runs off the page and the conclusion of every test is that the result was extremely statistically significant. The biology hasn’t changed. The questions haven’t changed. But increasing the sample size has guaranteed the same answer to every question.

## No-effect testing is imploding.

Debate over the problems with p-values constitutes a literature unto itself and cannot be reasonably covered here ^16,25 20,21,24 26,27 1,28 29^. The urgency of rehashing this debate for cell biology arises from three issues. 1) The misuse of testing for no-effect dominates quantification in cell biology. 2) Distinctions between important and trivial effects are critical to analyzing biological systems. 3) Automation means that the worst-case pitfalls of testing for no-effect are now a daily reality.

In the past, testing for no-effect often worked as a crude rule-of-thumb for effect size. If the sample sizes were small and measures were noisy, then a small p-value meant there was probably a big effect. But what happens to no-effect testing when automation allows massive datasets to be analyzed with minimum human supervision?

1. Large sample sizes drive p-values below threshold even when effect sizes are trivial.
2. Automation makes it possible to test thousands of conditions and variables that have a low average probability of being important. If follow-up experiments are evaluated with tests for no-effect, researchers will follow a drunkard’s walk^30^ from one trivial true-positive to the next.
3. Data analysis software makes it easy to generate many different highly derived measures from the same data. Interpreting the result with tests for no-effect makes it possible to claim a result is significant without anyone understanding what is being measured.

## Effect size should be at the center of planning, analyzing, and discussing experiments.

The reason testing for no-effect so profoundly distorts our science is that it allows us to analyze measurements without thinking about effect size. By effect size, I’m not referring to a specific statistical interpretation of effect size such as the standardized effect size (Cohen’s D^31^). By effect size, I just mean the magnitude of our measurements. For example, how big is the difference between two groups? What does that amount of difference mean for how the cells work? Effect size matters because: 1) Measurements are about effect size. 2) Effect size is how you judge the importance of an interaction. 3) Models of cellular processes run on effect sizes. 4) Effect sizes, and not statistical significance, can be compared between experiments. 5) Consideration of effect size, and not statistical significance, determines whether a field of inquiry is quantitative. Currently, it is difficult to argue that cell biology is operating as a quantitative science.

**Figure 1:**
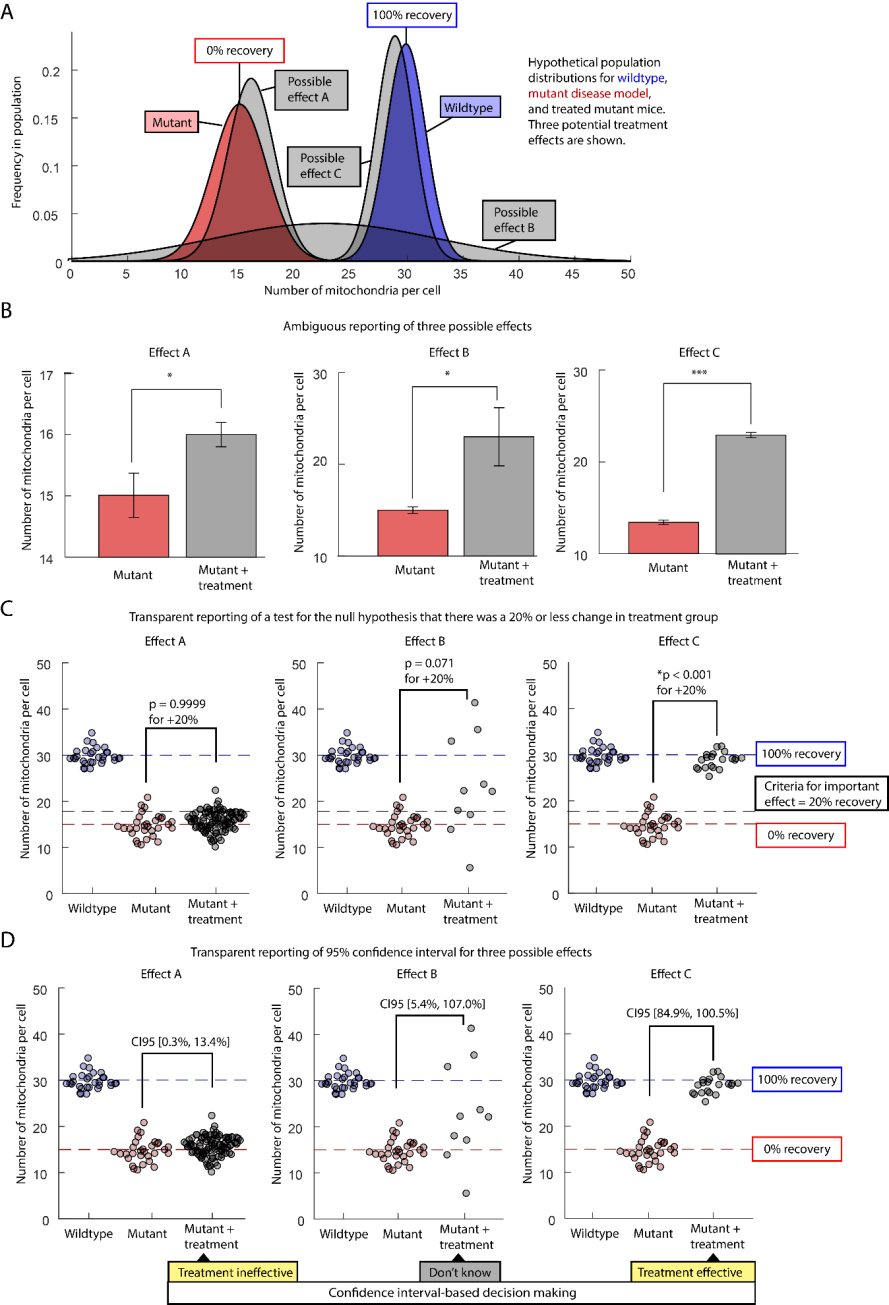
Comparison of hypothetical population distributions with different ways to represent results. Mitochondria number per cell is measured in healthy mice, a mutant mouse disease model, and in disease model mice that have undergone a treatment. Three possible results are shown for the treatment. A) Hidden population distributions of mitochondria number healthy (blue), disease model (red), and treatment (grey). B) All treatment results are statistically significant when the no-effect hypothesis is tested. C) Only result C is statistically significant when the null hypothesis of 20% or less recovery is tested. D) Confidence intervals are used to report effect size. Comparison in of groups indicate 95% confidence interval.

Considering effect size means that, prior to performing an experiment, we should be able to define how a measure maps onto biological meaning. Ideally, we will have at least one real-world example of a small effect and one real-world example of a important effect. Imagine we have a mutant mouse model of a disease in which the number of mitochondria in each cell is reduced to half. We want to use this mouse model to test the efficacy of a drug treatment. The data we collect is the number of mitochondria, but don’t necessarily care whether a cell has 20 or 21 mitochondria. We care about recovery. We, therefore, define a scale with mutant mitochondria number at one end (0%) and healthy mitochondria number at the other end (100% recovery = [treatment-mutant]/[healthy – mutant] * 100, Figure 1A). When we talk about our results, we present both the raw mitochondria number and the percent recovery.

Next, lets consider how we might analyze the results of a study of these mutant mice. The study will compare healthy mice, mutant mice, and treated mutants. Our worst option for statistical analysis is to put off thinking about statistical analysis until we have the data in hand. We then perform the default t-test for no-effect. If the null hypothesis is rejected, we accept the alternative hypothesis and conclude that the treatment was effective. Upon publication, we report our results with a series of bar graphs and asterisks (Figure 1AB). By treating a weak version of NHST as a rigorous test of a research hypothesis, we violate principles of science, statistics, and common sense. Critically, the dichotomized p-value quantification fails to distinguish between a trivial effect, an ambiguous result, and a complete cure (Figure 1AB). A reader who wants to determine if the treatment effect is biologically meaningful will have to decode the y axis of the plot, compare it to the standard error bars, then look up the sample size, and then re-read the text to try to determine what an important effect size might be.

A second analysis option is to insist on reporting a p-value, but to perform every step required for the p-value to inform a rational decision making process. Before performing the experiment, we formulate a quantitative model of the potential effects of treatment. This model incorporates existing quantifications of the efficacy of alternative treatments, how mitochondria number relates to health, potential sources of experimental bias, and incorporates a range of uncertainty about unknown variables. Based on this information, we propose a quantitative null hypothesis: the treatment will result in less than 20% recovery of mitochondria number. We then define a p-value threshold (p < 0.01) that reflects the relative costs of following up on a false positive result or neglecting to follow up on a false negative^13^. We perform a power analysis to ensure that our sample size could detect the effect size of interest. We preregister the experiment to reduce the impact of publication bias. Finally, we perform the experiment and calculate a one-tailed p-value. Given the totality of data and experimental design, this p-value gives us a quantitative and rational basis for making decisions. To report the results, we use scatter plots that show both the raw mitochondria counts (Figure 1C, y-axis) and our criteria for meaningful recovery (dashed lines). It is now clear to the reader what we consider an important effect size and how the data is distributed relative to that effect size.

To be clear, I am not familiar with a cell biology study that performed all the above steps for rigorous NHST. The above framework might make sense for a large-scale study that includes professional statisticians and that ends with a binary decision about how to proceed. The point of detailing the steps required to make NHST make sense, is that NHST is NOT the appropriate framework for what most cell biologists do most of the time. NHST is a particularly bad match for basic science experiments where the goal is not to inform binary decision making, but to figure out what is going on.

A third analysis option is to try a statistical framework that fits the kind of experiments we are usually able to do. Before the experiment we don’t know enough about the relevant effect sizes and variances to design a rigorous NHST or even formulate a quantitative (non-nil) hypothesis. The alternative to rigorous NHST is not to base conclusions on a weak NHST. The alternative is to rigorously characterize the effect size. We therefore calculate a confidence interval for the difference between the untreated mutant group and the treated mutant group (Figure 1D). When expressed in the form of percent recovery (84% to 100%), the confidence interval provides biologically meaningful information about whether the treated group looks more like the disease model mice or more like healthy mice. It is now easy to distinguish between experimental results that provide a precise estimate of an important effect size (CI95[97% to 99%]) and results that are compatible with both negligible and important effect sizes (CI95[5% to 200%]). Even if we did start with all the information required for a rigorous NHST, it is the confidence interval and not the p-value, that would provide useful information for understanding the biology and for comparing experiments.

## The confidence interval should be biology’s default summary statistic.

**Figure 2.**
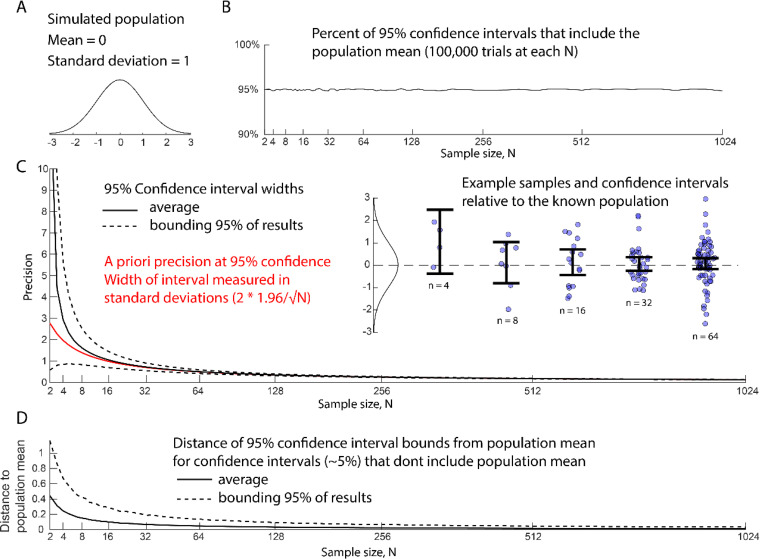
Performance of a standard 95% confidence interval when estimating the mean of a simulated normal distribution. Each sample size was tested with 100,000 samplings from the simulated population. A) Simulated population parameters. B) Percent of 95% confidence intervals that include the known population mean. The interval performs close to the nominal value. C) Width of confidence interval (precision) relative to sample size. Black line indicates the average width of the confidence interval. Dotted lines bound confidence interval width of 95% of trials. Red line shows the 95% a priori precision displayed as fractions of standard deviations. Inset shows example trials and confidence intervals relative to the sampled population. D) The confidence intervals that don’t include the population mean still approach the population mean as sample size increases. Traces are for trials where the confidence interval did not include the population mean. The black line is the average distance of the population mean to the nearest boundary of the confidence interval. The dotted line is the distance between the population mean and confidence interval that includes 95% of trials.

For any sample statistic (ex. mean = 10%), we can estimate a range of the population statistic that is most consistent with our data (CI95[8%,12%]). The goal of a given procedure for calculating a 95% confidence interval is that, if the same procedure was performed an infinite number of times, the real test parameter will fall within the bounds of the interval 95% of the time (Figure 2AB). As with p-values, the extent to which a given procedure achieves this performance depends on how well the statistical model fits the experiment ^32,33^.

Compared to p-values, confidence intervals are an efficient and transparent approach to reporting both effect size and precision (Supplementary Method 2). Confidence intervals respond in an intuitive way to how much information is available. More data means, on average, greater precision of effect size estimation (Fig 2CD). As a default statistic, confidence intervals have a huge advantage in that they provide useful information across wide range of experimental designs including analysis of preliminary data, rigorous descriptive studies, qualitative hypothesis testing, and quantitative hypothesis testing. Given these benefits, confidence intervals are the most commonly proposed alternative to p-values ^24,34–36^.

Confidence intervals are a good way to evaluate preliminary or exploratory data because they can be applied in the absence of a hypothesis and without any prior information about the magnitude of the variance or effect size. Calculating a confidence interval does require a model of variance and the precision of the estimates will be limited for small sample size (Figure 2CD)^37^. Note that at very low sample sizes, the precision of the confidence interval varies wildly. At these sample sizes, reporting a mean and a priori precision^38^ (Figure 2C,red line, see below) may be a more useful way to discus results. Even given the uncertainty of model fitting and noisy precision, calculating a quick confidence interval is reasonable on-the-fly tool for thinking about data. In contrast, consider the confused lab meeting conversations where students try to talk about five data points produced by new kind of measurement in terms of statistical significance and failing to reject the null hypothesis.

The most important function of confidence intervals is that they can be used to rigorously estimate effect sizes. Effect sizes are what matter for making decisions, interpreting biological significance, and building models. Cell biology often treats effect size estimation size as a lesser form of science than hypothesis testing. This bias seems to stem from confusion about the differences between descriptive science, descriptive statistics, hypothesis driven science, and statistical hypothesis testing. It is debatable whether most science is, or should be, hypothesis driven or question driven^39^. However, it is not debatable that testing a research hypothesis is not the same thing as testing a statistical hypothesis. If the goal is to test a research hypothesis, both effect size estimation (confidence intervals) and NHST (p-values) can provide relevant information. In the absence of a well-formed quantitative hypothesis, confidence intervals provide much more relevant information.

Even if a researcher starts with a quantitative hypothesis, confidence intervals are a useful summary statistic for interpreting results. A convenient symmetry to standard confidence intervals is that values outside of their bounds can be treated as hypotheses that are rejected at the given confidence threshold. Therefore, the information typically reported as “statistically significant” is readily available by checking if a null hypothesis is outside the confidence interval. More importantly, the confidence interval shows how far the null hypothesis is from the estimated range of effect sizes.

A confidence interval whose closest bound is far from the null hypothesis argues that there is something going on with the cells that is not adequately described by the null hypothesis. A confidence interval that includes the null hypothesis, or that is very close to the null hypothesis, argues that null hypothesis can predict most of effect of interest. Note that the distinction between “far from the null hypothesis” and “close to the null hypothesis” depends entirely on the biologist’s understanding of the measurement. There is no, and there can be no, single statistical criterion for evaluating a result as biologically meaningful.

Confidence intervals are sometimes criticized for setting arbitrary criteria (usually 95%) for defining a range. In contrast, p-values can report a continuous confidence value (p = .000341). That comparison leads us to the simple fact that confidence intervals and p-values are two sides of the same statistical coin. They are two ways to summarize the same statistical predictions. The difference between the two summary statistics is in which part of the statistical prediction is binarized. You can report a p-value, the precise fraction of a probability distribution defined by two magnitudes (null hypothesis and observed result). Alternatively, you can report a confidence interval, two magnitudes corresponding to a defined fraction of a probability distribution. In favor of reporting confidence intervals is the fact that there is no clear way to think about what a precise fraction of a hypothesized probability distribution means for our understanding of the world. The widespread confusion about p-values and probability reflects not just a lack of statistical sophistication, but also a genuine epistemological conundrum. We are on much more solid ground when we calculate a confidence interval and just treat the 95% as part of the statistical a procedure for calculating a useful estimate.

**Figure 3.**
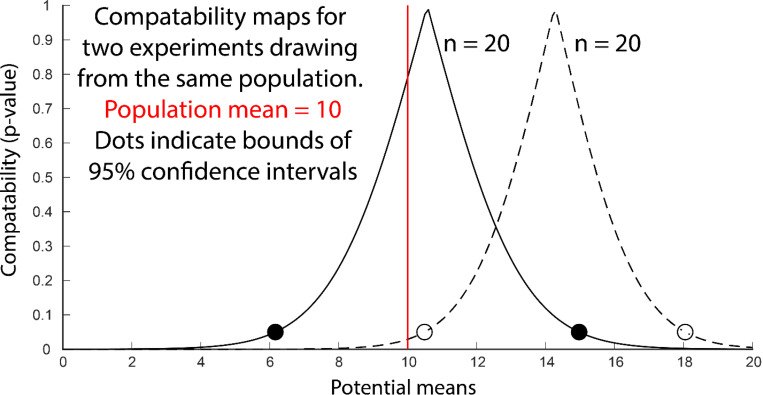
Compatibility maps produced by two simulated experiments (solid line and dotted line) that sampled from the same population. Each trace shows the p-value (y-axis) that would be produced by testing the experimental results against a range hypotheses (x-axis) about the population mean. The simulated population had a mean of 10 (red line) and a standard deviation of 10. The 95% confidence intervals are indicated by the dots.

It is possible to visualize the full range of a model’s predictions without imposing the threshold required for a confidence interval. A compatibility curve is a two-dimensional plot of the p-values (y-axis) that would be obtained for a range of plausible hypotheses (x-axis) given the observed data and a statistical model^40–42^ (Figure 3). The example shows the compatibility curves generated by two simulated experiments (n=20) that estimate a population mean using t-distributions (a bunch of t-tests). The two points where the compatibility map intersects p = 0.05 are equivalent to the bounds of the 95% confidence interval. Note, that the peak of each compatibility curve (p = 1) is simply the mean of the experimental sample. There is no additional hidden statistical magic that suggests that these peaks are “probably” the true mean. Reporting the results of a statistical model with a compatibility curve is especially useful for non-normal distributions that are not readily summarized by confidence intervals.

Summarizing data with confidence intervals does not guarantee statistical rigor. Because they are inferential statistics, confidence intervals are subject to many of the same assumptions and misinterpretations as p-values ^43,44^. If confidence intervals do become the default summary statistic in cell biology, we can expect to see similar overinterpretations of what confidence intervals can tell us about our data^11^. We can also expect effect sizes to be systematically inflated by measurement bias^45,46^, publication bias^47^, and the winner’s curse^48,49^.

One form of measurement bias warrants special attention because it often results from an attempt objectively analyze large and complicated data sets (omics). The researcher performs a manipulation experiment with thousands potentially interesting dependent variables (thousands of voxels, RNAs, genes, or cells). They set a criterion for identifying variables that were affected by the manipulation and then measure the size of the effect for those variables. The problem comes with the variance of the criterion for selection is not independent from the variance of the effect size being measured. Selecting variables based on their p-value, for instance, means that the same random fluctuations that push some variables above the selection criterion are also incorporated into the effect size calculation.

Ironically, the more conservative the selection criterion (p < 0.00001), the more inflated the apparent effect sizes will be^49^. The solutions include defining an independent criterion for selection^46^ or perform an adjustment to the effect size estimate that takes the selection criterion into account^50^.

Even when properly acquired and interpreted confidence intervals are not the ideal statistic for every experiment. For example, as a measure of precision, confidences intervals lump together different classes of error (measurement precision, variation within the population, and sampling error) that could be calculated and reported separately^51^. More generally, statisticians point out that statistical techniques are constantly being improved and that it is wrong headed to expect one type of analysis to be optimal, or even appropriate, for most experiments^1^. Given all these limitations, why is it worth pushing tens of thousands of cell biologists to switch to calculating confidence intervals?

I am not arguing that confidence intervals should be cell biology’s default method of analyzing data. Obviously, the default method of analyzing our data should be staring at scatterplots^52^. What I am arguing is that confidence intervals should be our default *summary statistic*. NHST for no-effect became ubiquitous because there is a genuine niche for a simple and universally understood quantification of what we see when we look at scatterplots (Supplementary Method 3). Reporting p-values was a reasonable idea, but misuse and misinterpretations proved disastrous. Replacing p-values with confidence intervals would, at least, mean that cell biologist would start talking about their measurements in terms of effect size and precision.

Imagine again the dial that increases sample size in all publications. This time, the publications base their claims on confidence intervals. As sample sizes increase towards infinity, the intervals shrink to point values that are the actual value of the population. When effect size estimation is the foundation of our analysis, more data translates into more understanding.

## Philosophical problems with trying to quantify inference

Ignoring p-values and replacing them with confidence intervals is a pragmatic solution to the current quantification crisis in cell biology. The crisis is that cell biologists have learned to talk about biology without talking about effect size. Confidence intervals can fix this problem without requiring us to make changes to how we perform experiments or requiring much additional statistical sophistication. But teaching students to calculate zombie confidence intervals is not the ideal end point for the statistical maturation of a field^27^. Ideally, we would teach cell biologists that quantification and statistical analysis are part of the process of thinking about biology and not just a checkbox for publication.

As human beings and scientists we theorize there is a universe that we collect information about through our experiences. From these experiences we build mental models of bits of the universe (inference) that make predictions about future experiences. If the universe works like X, we can expect Y to happen. If these predictions are inaccurate, we refine or reject the model. A statistical model is an abstracted version of a mental model whose behavior can be described mathematically. The brilliance of these statistical models is they can be applied by many different observers, to many different processes, and that their predictions are repeatable and quantifiable. However, the extent to which the model tells you something useful about the universe depends on how well each assumption of the model corresponds the behavior of the patch of the universe you are investigating.

The quantifications of frequency and probability produced by a statistical model, therefore, are only quantitative as statements about that model. The 95% of the 95% confidence interval is about a set of modeled distributions. The 95% is not about whether the underlying model is appropriate to the question or whether there was a bias in your sampling procedure. The extent to which the numbers from statistical analysis can be extrapolated to beliefs about the universe is a challenging practical and epistemological problem. Bayesian statistics improves the situation by being explicit about the landscape of probability distributions we expect prior to performing our experiment ^44^. However, these prior probabilities are also not immune to the uncertainties about how well our models and prior beliefs fit reality. Ultimately, no matter how small our p-value or confidence interval is, there is still about a one in four(?) chance we did the experiment wrong.

We are on philosophically safe while we are thinking about statistical inference as thought experiments instead of facts about the world^17^. However, a useful cognitive model of the world requires that we eventually summarize evidence as something like: “most cell bodies are probably about 10 μm in diameter”. Statistical analysis can help us fill in the blanks for ‘most’, ‘probably’, and ‘about’, but the relationship can’t be one-to-one. It is up to the researcher and reader to decide how much the experiment, results, and statistical model should inform their belief about the biology.

One path out of these philosophical ambiguities is to focus on statistics as a tool for designing experiments rather than for quantifying inference. Trafimow (reviewer) argues for a shift from post hoc data analysis to an a priori procedure in which the researcher predefines a desired level of precision and desired probability of achieving that precision^38,43^. If a distribution is treated as normal and precision is measured in units of standard deviations, the standard deviations cancel out and the appropriate sample size can be calculated for any combination of precision and confidence. For instance, if I want to design an experiment with a 95% probability (z-score = 1.96) that the measured mean will be within ±0.5 standard deviations of the population mean, I need a sample size of 16 (*N* = (1.96/.5)^2). This calculation avoids the confounding estimate of population variance that is built into a confidence interval. The math of calculating sample size for a given target precision (and the reverse: precision = 1.96/√*N* ) is, therefore, a useful tool for designing and describing cell biology experiments. The limitation of this approach is that it requires researchers to think about precision in terms of multiples of unknown standard deviations. Translating data into an understanding of cellular mechanisms will, therefore, usually still benefit from a statistic like the confidence interval that can report precision in terms of the unit of measurement.

A second path out of the philosophical confusion is for more cell biologists to perform statistical analysis by programming Monte Carlo simulations^53^. Programming these simulations allows a researcher who is unfamiliar with the fine points of statistical theory to ask their question in the most straightforward way possible: “If the system worked like this and I ran my experiment 100,000 times, what would I expect to see?” The act of writing the simulation forces the researcher to think of the statistical model as a set of assumptions and “if, then” statements that may or may not match reality. It could also push the researcher to question whether a hypothesis they are simulating is really a plausible way for the system to work. At the very least, a student who calculates p = .00013 as a frequency in a simulation they just programmed should be less likely to believe that means there is a 13 in 100,000 chance that the null hypothesis is true.

Finally, cell biologists should recognize statistics as a field study and not a set of inherited facts. Improved methods of inference and error analysis are actively being developed.

## What happens to cell biology if we stop reporting tests for “no-effect”?

In effect-size-centric cell biology, the gold standard for quantification would be to report estimates of effect sizes that reliably distinguish between plausible models of the system. Reliability would be judged by both the precision of the measurement and by experimentally independent verification of the result. The criteria of “biologically meaningful effect size” is a much higher bar than “statistically significant”. Under this criterion, many fewer studies can be expected to claim important experimental effects. This aspect of increased statistical rigor does not necessarily mean fewer papers will be published. An unbiased literature requires publishing both “positive” and “negative” results. The practice of pre-registering experiments with journals or online databases and then reporting results regardless of the outcome not only reduces p-hacking, but also reduces distortions of effect sizes^47^.

Focusing our analysis on effect size does mean that we will have to accept the reality that most experimental results are ambiguous and incremental. The implicit and, sometimes, explicit interpretation of p-values is that the result of every experiment is either “effect” or “no-effect”. When we take confidence intervals seriously, we will often have to conclude that we don’t have enough data to understand what is going on.

Reporting and interpreting confidence intervals will also require more work than reporting tests for no-effect. It is extremely convenient for everyone to agree that there is a statistic (p-value) that tells you that something important happened even when it is not clear what is being measured. In the absence of this fiction, our claims will require more explanation about how our measure relates to the biology and how the observed effect size relates to our question.

Arguing that we should produce a more detail-oriented literature filled with ambiguous and negligible effect sizes is not the most appealing pitch for changing the way cell biology quantifies data. What we could gain is a literature in which biological claims reflect meaningful quantification, a literature from which we can judge the replicability of experiments, and a literature from which we can build better models of how cells work.

## Supplementary Material

Supplement 1
