# Supplementary material for: Alternatives to the statistical mass confusion of testing for “no effect”
Source: ArXiv. 2025 Apr 7:arXiv:2407.07114v2. Preprint. [Version 2] (PMC12036429)
Supplement: Supplementary file 1 [file NIHPP2407.07114v2-supplement-1.pdf]

SUPPLEMENTAL METHODS

1) Comparing an experimental effect to a Monte Carlo distribution

Imagine we want to know if synaptic inputs to a neuron are concentrated on the apical dendrite of some class of neuron. We perform an experiment where we find that the densities of synapses on the apical dendrites are about twice as high as the densities on the rest of the cell. What we want to know is if this discrepancy can be explained by how often the apical dendrite encounters potential synaptic partners. We, therefore, build a Monte Carlo simulation of the tissue that includes complicated anatomical constraints such as the geometry of the cells, the number of synapses, and the distances between synapses. We run the model 100,000 times and produce a distribution for the predicted densities of synapses on the apical dendrite. How do we compare our observed results to the Monte Carlo model?

We could generate a p-value by counting how many of the results have an equal or greater density of synapses on the apical dendrite. A very low p-value might support the argument that the observed bias would be unlikely to be produced by the anatomy as simulated. But what if we forgot to factor in subtle changes in the diameters of dendrites? What if the density of blood vessels is different for different regions of the tissue and they obstruct some opportunities for making synapses? A small p-value could also result from any number of trivial anatomical biases that were not controlled for by the model.

The question we should care about is how much of the bias in synapse density can be explained by a reasonable set of anatomical constraints. To make this comparison, we calculate the range of values that encompass 95% (or 90% or 99.9%) of the Monte Carlo results. We can then compare these bounds to our observed bias. One possible result is that the density of synapses we observe on the apical dendrite (400/mm) is far outside of the predicted range (CI95[150/mm, 250/mm], Supplementary Figure). In this case, we can now use the upper bound of the Monte Carlo range to report a conservative estimate of the bias towards forming synapses on the apical dendrite: “The observed apical dendrite density was at 60% higher than the upper bound predicted by our model”. We might also find that our observed density is within the range predicted by our model. In that case, it is useful to report if inclusion of the observed value is because the model precisely predicts the density (CI95[390/mm, 410/mm]) or because the model predictions include a wide range of outcomes (CI95[50/mm, 800/mm]). Regardless, of the result, a histogram of the experimental results plotted against the Monte Carlo results provides the most transparent description of the results (Supplementary Figure).

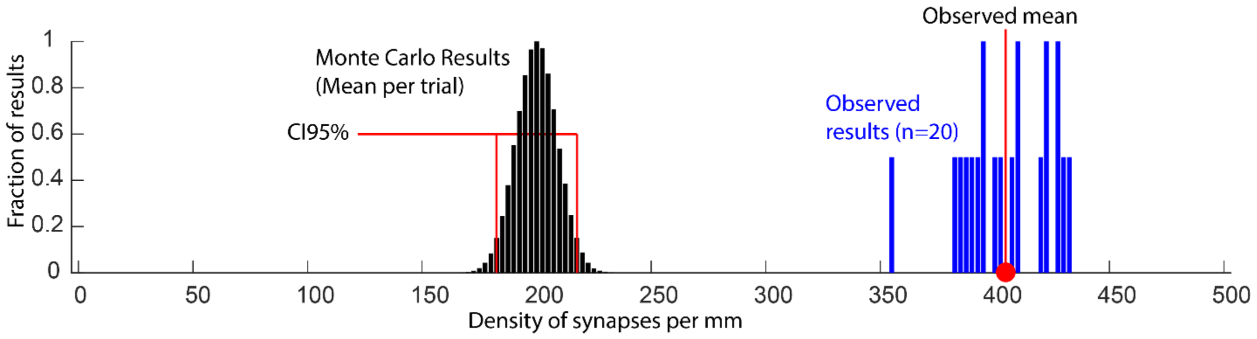

Supplementary Figure. Comparison of observed results to the results of a Monte Carlo simulation. The means of 100,000 Monte Carlo simulations are shown in black. The bounds of the 95% confidence interval encompassing these results are bracketed. The observed sample is shown in blue with the mean highlighted with the red circle. The conservative estimate of effect size is the distance between the red circle and the nearest CI95 bracket.

2) Calculating confidence intervals.

There are a variety of methods for calculating confidence intervals and many of these are built into common statistical software packages. The goal when choosing a method is to find one for which the actual coverage is as close to possible to the nominal coverage. If you calculate a 95% confidence interval for the mean of a distribution, the nominal coverage is 95%. The claim the calculation is making is that, 1) if the assumptions of the model are met and 2) if the same calculation was repeated many times for similar datasets, then 3) the calculated interval would contain the true mean 95% of the time. The extent to which this claim is true, the actual coverage of your interval, depends on how well the assumptions of the calculation fit the data.

When calculating a confidence interval to compare two groups, the model assumptions to consider are roughly the same as when calculating a p-value: Are the distributions normal? Are the sample sizes the same? Are the sample sizes large enough for a given model? Are the variances the same? Are the distributions skewed or asymmetric? As in calculating p-values, non-parametric resampling techniques can be used that make very few assumptions about the distribution of the data[2–8]. For instance, generating a confidence interval with bootstrap resampling is a common approach. For a default statistic for cell biology though, the most robust and practical approach is to start with the same statistical model we are used to using for t-tests and then to adjust as required.

The simplest way to generate a confidence interval for the difference in means between two groups is to pool the standard errors (pooled SE = sqrt(SE1^2 + SE2^2). The 95% confidence interval is then the difference between the two groups plus and minus the pooled standard error multiplied by 1.96. This method is convenient when calculating a back of the envelope confidence interval from published standard errors, but it is an approximation that

assumes that sample sizes are large and sample sizes and variances are equal between the groups.

The Welch-Scatterhwaite confidence interval was designed to use the t distribution to calculate confidence interval when the variances of two groups are different [9]. Miao and Chiou[10] find it performs well when sample sizes are the same or different, when variances are the same or different, and even when distributions are not normal. They do find that its performance decreases when distributions are asymmetric and therefore recommend a pretest for symmetry and log transformation of asymmetric data. With the caveat of detecting and correcting for asymmetric distributions, the Welch-Scatterhwaite confidence interval is then a single calculation that could reliably replace most of the p-values in the cell biology literature.

The Welch-Scatterhwaite confidence interval is more complicated to calculate than the pooled variance interval [10], but it is an option in most statistics software packages. For example in Matlab 2023a the WS interval can be obtained by the expression: [noThanks1 noThanks2 confidenceInterval] = ttest2(group1, group2, “Alpha”, 0.05, “vartype”, “unequal”). To experiment with how different kinds of confidence intervals interact with different kinds of data, Matlab code is available for download (https://github.com/MorganLabShare/betterThanChance).

<u>Calculating the Welsh-Scatterhwaite confidence interval for the difference between two groups</u>

1. Calculate the combined standard error (sp) using the standard deviation (std1, std2) and sample size (n1,n2) for two groups: $w1 = \frac{std1^2}{n1}, w2 = \frac{std2^2}{n2}, sp = \sqrt{(w1 + w2)}$
2. Calculate the degrees of freedom (df): $df = (w1 + w2)^2/(\frac{w1^2}{n1-1} + \frac{w2^2}{n2-1})$
3. Look up the t-score (t) for the desired alpha using the t-distribution with df degrees of freedom. For a 95% confidence interval, alpha = 0.05: $t = tDistribution([1 - \frac{alpha}{2}], df)$
4. Calculate the confidence interval as the difference between means (m1,m2) plus and minus the combined standard error (sp) scaled by the t-score (t): $CI = (m1 - m2) \pm t * sp$

REFERENCES

1. Krantz, D.H. (1999). The Null Hypothesis Testing Controversy in Psychology. J. Am. Stat. Assoc. *94*, 1372–1381.

2. Efron, B. (1979). Bootstrap Methods: Another Look at the Jackknife. aos *7*, 1–26.

3. Fieberg, J.R., Vitense, K., and Johnson, D.H. (2020). Resampling-based methods for biologists. PeerJ *8*, e9089.

4. Jung, K., Lee, J., Gupta, V., and Cho, G. (2019). Comparison of Bootstrap Confidence

Interval Methods for GSCA Using a Monte Carlo Simulation. Front. Psychol. *10*, 2215.

5. Shi, S.G. (1992). Accurate and efficient double-bootstrap confidence limit method. Comput. Stat. Data Anal. *13*, 21–32.

6. Letson, D., and McCullough, B.D. (1998). Better Confidence Intervals: The Double Bootstrap with No Pivot. Am. J. Agric. Econ. *80*, 552–559.

7. Puth, M.-T., Neuhäuser, M., and Ruxton, G.D. (2015). On the variety of methods for calculating confidence intervals by bootstrapping. J. Anim. Ecol. *84*, 892–897.

8. DiCiccio, T.J., Martin, M.A., and Young, G.A. (1992). Fast and accurate approximate double bootstrap confidence intervals. Biometrika *79*, 285–295.

9. Welch, B.L. (1938). The Significance of the Difference Between Two Means when the Population Variances are Unequal. Biometrika *29*, 350–362.

10. Miao, W., and Chiou, P. (2008). Confidence intervals for the difference between two means. Comput. Stat. Data Anal. *52*, 2238–2248.
